# Impact of customized electronic duplicate order alerts on microbiology test ordering: Financial and environmental cost savings

**DOI:** 10.1017/ice.2023.198

**Published:** 2024-03

**Authors:** Maryza Graham, Robert Gugasyan, Devisri Dharmaraj, Gillian Yap, Brooke Webb, Anjali Dhulia, Beena Kumar

**Affiliations:** 1 Department of Microbiology, Monash Health Pathology, Monash Health, Clayton, Victoria, Australia; 2 Monash Infectious Diseases, Monash Health, Clayton, Victoria, Australia; 3 Faculty of Medicine, Nursing and Health Sciences, Monash University, Victoria, Australia; 4 Victorian Infectious Diseases Reference Laboratory, Peter Doherty Institute for Infection and Immunity, Victoria, Australia; 5 Monash Health Pathology, Monash Health, Clayton, Victoria, Australia; 6 Office of Chief Medical Officer, Monash Health, Clayton, Victoria, Australia; 7 Chief Medical Officer, Monash Health, Clayton, Victoria, Australia

## Abstract

**Objective:**

To estimate cost savings after implementation of customized electronic duplicate order alerts.

**Design:**

Alerts were implemented for microbiology tests at the largest public hospital in Victoria, Australia. These alerts were designed to pop up at the point of test ordering to inform the clinician that the test had previously been ordered and to suggest appropriate reordering time frames and indications.

**Results:**

In a 6-month audit of urine culture (our most commonly ordered test) after alert implementation, 2,904 duplicate requesters proceeded with the request and 2,549 tests were cancelled, for a 47% reduction in test ordering. For fecal polymerase chain reaction (PCR), our second most common test, there was a 54% reduction in test ordering. For our most commonly ordered expensive test, hepatitis C PCR, there was a 42% reduction in test ordering: 25 tests were cancelled.

Cancelled tests resulted in estimated savings of AU$52,382 (US$33,960) for urine culture, AU$34,914 (US$22,442) for fecal PCR, AU$4,506 (US$2,896) for hepatitis C PCR. For cancelled hepatitis B PCR and Epstein-Barr virus (EBV) and cytomegalovirus (CMV) serology, the cost savings was AU$8,472 (US$5445). The estimated financial cost saving in direct hospital costs for these 6 assays was AU$100,274 (US$67,925) over the 6-month period. Environmental waste cost saving by weight was estimated to be 280 kg. Greenhouse gas footprint, measured in carbon dioxide equivalent emissions for cancelled EBV and CMV serology tests, resulted in a saving of at least 17,711 g, equivalent to driving 115 km in a standard car.

**Conclusion:**

Customized alerts issued at the time of test ordering can have enormous impacts on reducing cost, waste, and unnecessary testing.

Diagnostic stewardship aims to influence the process of ordering, performing, and reporting diagnostic tests to improve patient care and reduce waste.^1^ The diagnostic stewardship approaches with the highest reported success rates include computerized clinical decision support (CCDS) interventions and real-time evaluations.^2^

Unnecessary repeat testing is a good target for diagnostic stewardship. Duplicate testing is valid in some instances but is often unnecessary. Similar to the experience of others, we have found that duplicate testing is often performed because the second ordering clinician is unaware of a previous order or is too busy to check the pending order list.^3^ Navigation away from the ordering screen to check for pending orders requires additional steps that may be time consuming. Duplicate laboratory tests have several potential adverse effects. Pain or discomfort may occur with repeated specimen collection. Iatrogenic anemia may occur from repeated phlebotomies, which in turn may affect wound healing and lead to infection. Increased healthcare costs can accrue through specimen collection, transport, testing, storage, and resulting waste disposal. The clinical response to the result may include unnecessary treatment or further investigation.^3^ Overdiagnosis of healthcare-associated infections may lead to inappropriate antimicrobial use and risks of antimicrobial resistance and adverse drug effects as well as their associated unnecessary costs.^4^

Repeat testing is associated with low diagnostic yield and with increased false-positive results. For example, *Clostridioides difficile* studies revealed that testing repeated within 48 hours has low diagnostic value.^2^ Additionally, in a study prompted by a pseudo-outbreak at an academic center, repeated testing led to an increase in false-positive results due to the decrease in prevalence: the positive predictive values of the second and third *C. difficile* tests were 30% and 4%, respectively.^5^ Repeated *C. difficile* polymerase chain reaction (PCR) testing has been associated with increased cost to the patient and healthcare system.^2,6–9^ Furthermore, test stewardship driven by electronic medical record (EMR)–based CCDS decreased the frequency of *C. difficile testing*, improved test fidelity, and decreased the number of patients potentially overtreated with antibiotics for *C. difficile* colonization.^10^

Electronic test ordering provides opportunities for pathology stewardship and the implementation of duplicate order alerts to decrease repeat testing. Few studies have measured the impact of reduction of duplicate orders on tests other than *C. difficile*.^11–13^ In one such study, an intervention to block duplicate orders demonstrated avoidance of 11,790 tests in 2 years, with cost savings of US$183,586. The CCDS intervention consisted of an immediate electronic notification alert that a same-day duplicate test was being ordered and informed the provider that repeat testing is not warranted more than once per day.^3^ In another study, implementation of a new order set in the electronic health record that required practitioners to choose an indication for the type of urine study, led to a 40% reduction in urine cultures performed, a decrease in antibiotic days of therapy, and substantial financial savings.^14^

The cost of infectious waste disposal is much higher than that of noninfectious waste. For example, in the United States, it costs US$0.79 per kilogram to dispose of infectious waste, which represents a 560% cost premium over the typical noninfectious waste disposal cost of US$0.12 per kilogram. It has been estimated that as much as 23% of overall infectious medical waste comes from laboratories.^15^ In a recent study of the entire New South Wales health system, pathology and diagnostic imaging together accounted for ∼9% of its healthcare footprint comprising total emissions, water use, and waste footprints.^16^ A recent Australian study was conducted to estimate the carbon footprint of 5 common pathology tests. These researchers concluded that reducing unnecessary testing would be the most effective approach to reducing the carbon footprint of pathology testing.^17^

Some electronic test-ordering systems allow design of customized duplicate order alerts with CCDS tailored to the test type. In our study, we implemented test-specific duplicate order alerts based on various repeat-test time frames (ranging from 3 days to 2 years), in which the alert message suggested time frames and clinical indications for appropriate retesting. We measured the impact of these customized alerts on ordering over a 6-month period. We studied the environmental impact of repeat testing given that climate change influences health outcomes and that requesters may be motivated to order tests prudently if they are more aware of the environmental impacts.

## Methods

Monash pathology laboratory services Monash Health, the largest public healthcare network in Melbourne, Australia, with 2,150 inpatient beds and 3 emergency departments serving more than one-quarter of Melbourne’s population. The laboratory also serves outpatient clinics and accepts pathology samples referred by local general practitioners.

Duplicate order alerts were implemented on October 15, 2020, for inpatients and Emergency Department patients at Monash Health. Data were extracted from the electronic medical record retrospectively from February 17, 2022, to August 17, 2022. We chose to study the 2 most commonly ordered microbiology tests (urine culture and fecal PCR) and serology tests [ie, Epstein-Barr virus (EBV) and cytomegalovirus (CMV) serology] and the 2 most commonly ordered expensive microbiology tests (hepatitis C and hepatitis B PCR).

We performed a budget impact analysis^18^ of direct financial cost savings to the hospital of implementing duplicate order alerts. We used the Australian Medicare Benefits schedule fee to determine cost savings for each test:^19^ urine culture (item 69333; AU$20.55 or US$13.21), fecal PCR which includes targets for *C. difficile* toxin (item 69496; AU$43.05 or $US27.67), hepatitis C PCR (item 69488; AU$180.25 or US$115.86), hepatitis B PCR (item 69482; AU$152.10 or US$97.77), EBV serology (item 69474; AU$28.65 or US$18.42), and CMV serology (item 69387; AU$29 or $US18.64).

We used specimen container volume to measure the waste disposal savings by weight: urine container (Sarstedt multipurpose container, 70 mL, 75.9922.721, 55 × 44 mm, 12 g), feces tube (Sarstedt 70 mL, 80.9924.027 55 × 44 mm, 13.5 g), hepatitis C and hepatitis B PCR (BD Vacutainer Plasma Preparation tube PPT, 5 mL, 362791, 13 ×100 mm, 8 g) and EBV/CMV serology (BD Vacutainer SST, 5 mL, 367954, 13 × 100 mm, 8 g).^20,21^ We estimated the weight of each urine or feces sample by assuming that each container with 70 mL water weighed 70 grams. The mean weight of 1 mL blood is 1.06 g, and we estimated that the weight of a full PPT or SST tube containing 5 mL blood was 13.3 g including the weight of the empty tube (Supplementary Table 1 online).^22^

We estimated greenhouse gas footprint, measured in carbon dioxide equivalent (CO_2_e) emissions. The CO_2_e attributed to sample collection consumables for EBV and CMV serology tests was based on previous calculations of 89 g per test for swabs, gloves, vacutainer holders, and collection tubes and specimen bags for Gold Top tubes. We estimated equivalent emissions produced by distance driven for which 1 g CO_2_e emissions produced equivalent emissions as 6.5 m travelled in a standard car.^17^

To determine the direct cost to the hospital of implementation of the duplicate order alerts, we reviewed information technology staff logs to calculate the time required for staff to configure the alerts and code behind them and to validate the functioning of the alerts.

**Figure 1.**
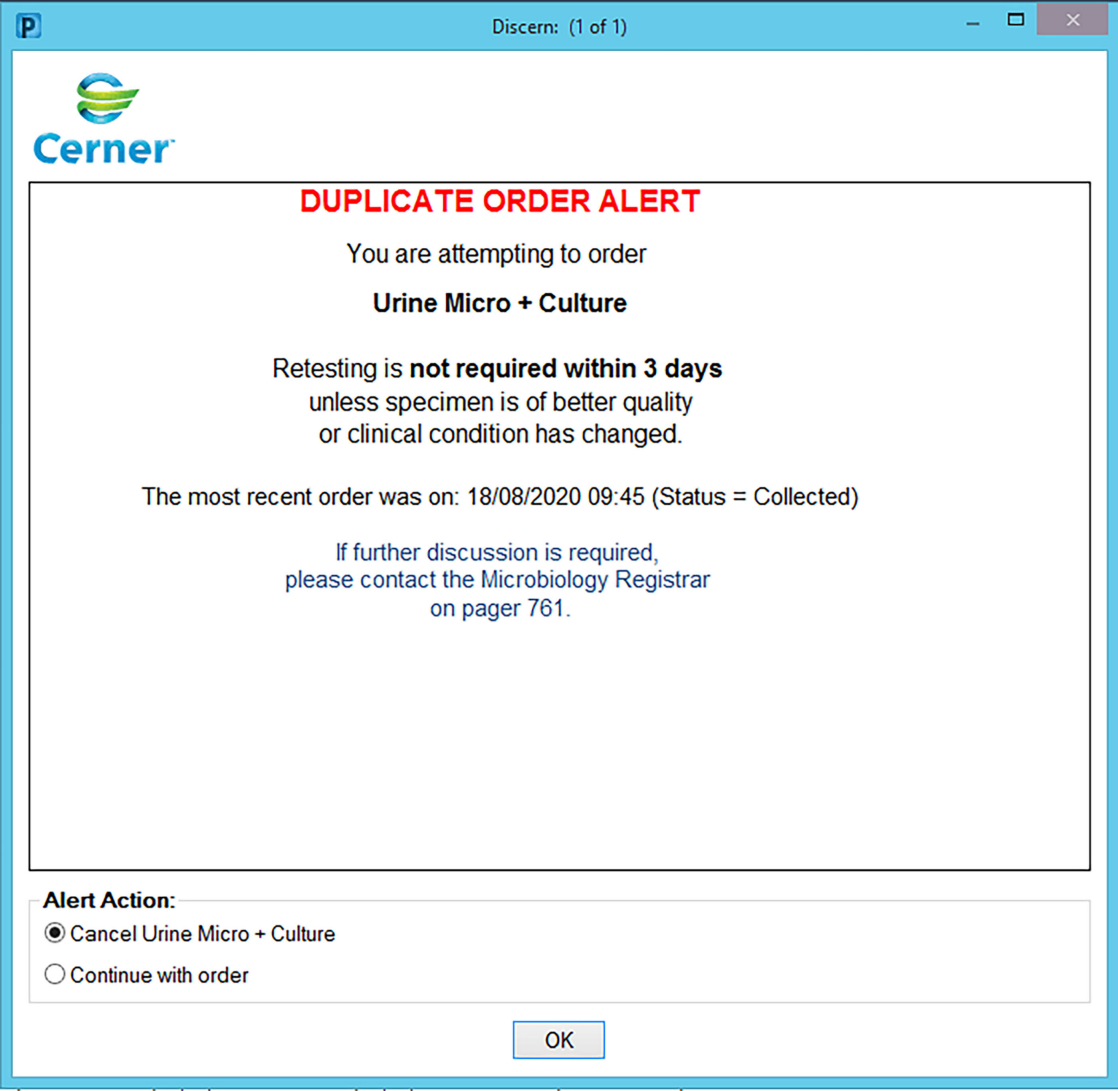
Example of duplicate order alert for urine microscopy and culture.

**Figure 2.**
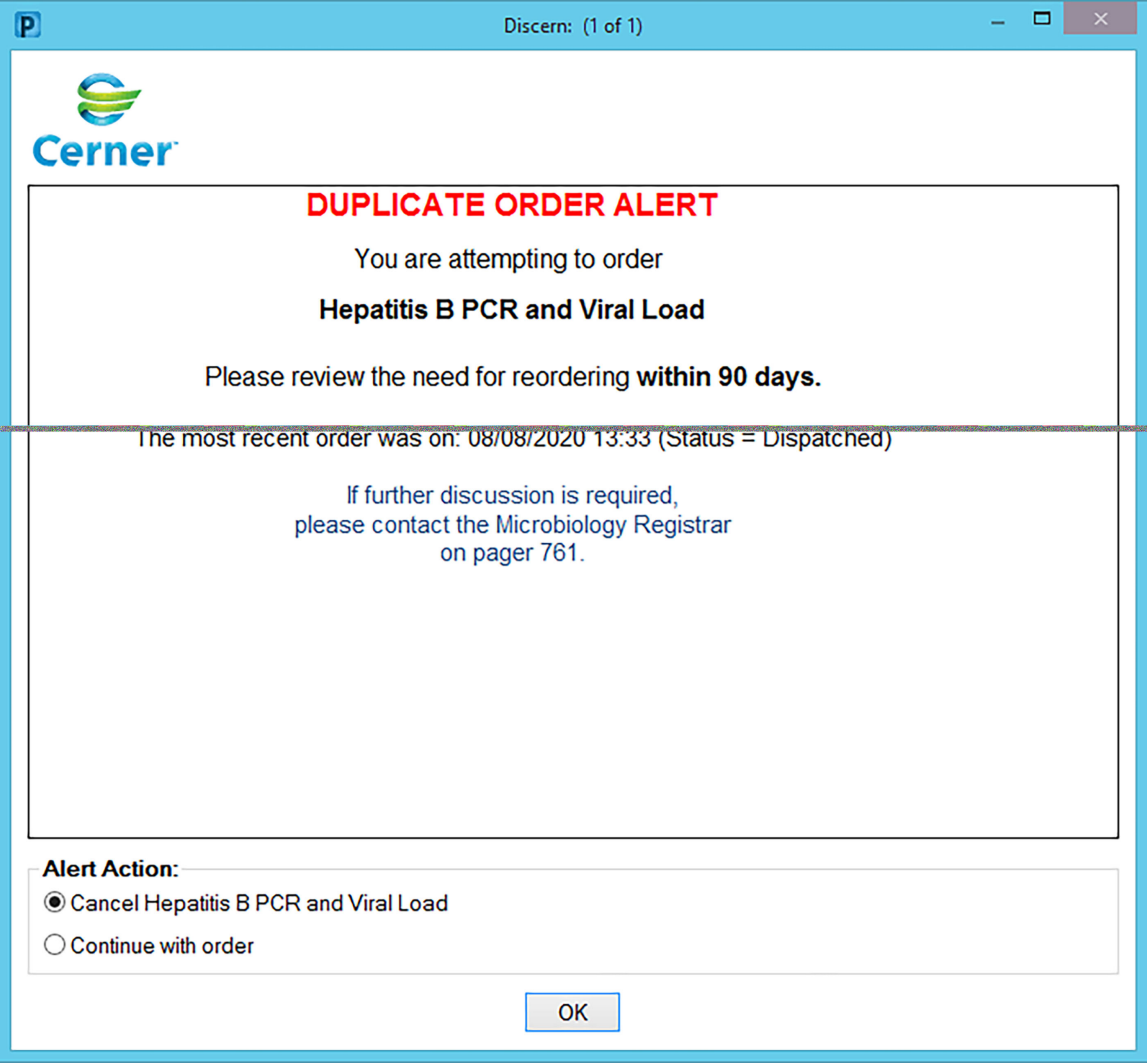
Example of duplicate order alert for Hepatitis B PCR.

We grouped tests based on appropriate time frames for repeat testing. These time frames (ranging from 3 days to 2 years) were based on national quality assurance and treatment guidelines, and Australian Medicare reimbursement time frames. They were agreed upon by medical microbiologists and senior scientific staff. Alerts were designed to trigger based on these test time frames. For each test group, we also formulated a customized comment to be included as an alert to guide the clinician’s decision making (Table 1). Examples of 2 different duplicate order alerts are shown in Figure 1 and Figure 2.

**Table 1.** Test Groups, Minimum Retest Intervals and Alert Text

Test Group	Minimum Retesting Interval	Alert Text
Group 1		
Urine micro + culture Sputum micro + culture	3 d	Retesting is not required within 3 days unless specimen is of better quality or clinical condition has changed.
Group 2		
EBV IgG and IgM serology EBV IgG serology CMV IgG and IgM serology CMV IgG serology Hepatitis A total Ab Hepatitis B core total Ab Human herpes virus 6 serology	7 d^a^	Retesting is not required within 7 days or if IgG has previously been detected.
Group 3		
Adenovirus serology Amoebic serology Angiostrongyliasis serology Anti-DNAse B titer Anti-Streptolysin O titer *Bartonella henselae* Ab *Bordetella pertussis* serology Barmah Forest virus serology Chikungunya serology Chlamydia/gonorrhoea/ trichomonas PCR Chlamydiophila serology Coccidioidomycosis serology Cysticercosis serology Dengue virus serology Diptheria toxoid IgG EBV PCR EBV viral load Faecal pathogen PCR	7 d^a^	Retesting is not required within 7 days.
Fasciola IgG Filaria antigen Filiarial antibody Flavivirus serology Galactomannan Hantavirus serology Hendravirus serology Hepatitis B and C serology (acute) Hepatitis B e antigen serology Hepatitis B serology (acute) Hepatitis B surface antibody Hepatitis B surface antigen Hepatitis C serology Hepatitis D serology Hepatitis E serology Herpes/CMV/Adenovirus PCR *Histoplasma CAPSULATUM* Serology HTLV 1 + 2 SEROLOGY Hydatid serology Japanese B encephalitis serology *Legionella* serology *Leishmania* serology Leptospirosis serology Mumps IgG and IgM serology *Mycoplasma pneumoniae* serology (convalescent) *P. westermani* serology Polyomavirus PCR Q-fever serology Rickettsia serology Ross river virus serology Schistosoma serology Strongyloides IgG serology *Toxocara canis* antibody *Trichinella spiralis* IgG *Trypanosoma cruzi* serology West Nile virus serology Yersinia serology Yellow fever serology Zika virus serology		
Group 4		
Hepatitis B viral load PCR Hepatitis B s antigen quantitative serology Hepatitis C viral load PCR	90 d^b^	Please review need for reordering within 90 days.
Group 5		
Hepatitis C genotype	2 y^b^	This test has been ordered within the last 2 years. Once a hepatitis C genotype has been determined repeat testing is rarely warranted.
Group 6		
*Mycobacterium tuberculosis* Rifampicin gene detection *Mycobacterium tuberculosis* PCR Respiratory sample	7 d^a^	Please discuss with microbiology registrar (pager no.) if retesting is required within 7 days.
Group 7		
HSV type 1/2 serology	7 d^a^	First-order decision support comment and duplicate order alert: This test is not routinely performed. The ideal method for definitive diagnosis of HSV infection is viral detection by PCR. Please send a flocked swab (or CSF/blood/tissue) for Herpes PCR, if clinically indicated. Herpes simplex serology testing will not be performed unless relevant clinical notes are provided. If further discussion is required, please contact the microbiology registrar.
Melioidosis serology		This test is not routinely performed. Culture is the mainstay of diagnosis of melioidosis and serology is rarely useful. Please send blood, sputum, urine, swab of ulcer/skin lesion, throat swab and rectal swab (as available) for culture and indicate “? Meliodosis” in the clinical notes. Melioidosis serology testing will not be performed unless relevant clinical notes are provided. If further discussion is required, please contact the microbiology registrar.
Polio serology		Poliovirus serology is only performed if there is a clinical indication of acute infection in a susceptible person. Poliovirus serology testing will not be performed unless relevant clinical notes are provided. If further interpretation is required, please contact the microbiology registrar.
Group 8		
Group B *Streptococcus* screen	5 wk^c^	Retesting is not required within 5 weeks unless specimen is of better quality or clinical condition has changed.

Note. Retesting intervals were based on the following references (see Methods).

a Royal College of Pathologists of Australasia (RCPA) Quality Assurance Program Survey Reports.

b Reference^19^. Medicare Benefits Schedule. Australian Government, Department of Health and Aged Care. Published July 2022. Accessed 8th February 2023. https://testingportal.ashm.org.au/treatment-of-chronic-hepatitis-b-virus-infection/. Accessed February 8, 2023. https://www.ashm.org.au/resources/australian-recommendations-for-the-management-of-hepatitis-c-virus-infection-a-consensus-statement/. Accessed February 8, 2023.

c https://ranzcog.edu.au/wp-content/uploads/2022/05/Maternal-Group-B-Streptococcus-in-Pregnancy-Screening-and-Management.pdf.

Ethics approval for the study was obtained from the Monash Health Research Office (no. RES-23-0000136Q-94743).

## Results

Pathology ordering practice was studied over the 6-month period from February 17, 2022, to August 17, 2022, which was 16 months after the duplicate alerts were implemented. For these 6 tests, 4,020 duplicate requesters proceeded and 3,602 tests were cancelled, resulting in a 47% reduction in test ordering (Table 2).

**Table 2.** Direct Cost Savings and Direct Costs to the Hospital

		Cost of Cancelled Tests			
Variable	No. of Cancelled Tests/Proceeded Tests	AU$	US$	Waste of Cancelled Tests, kg	CO_2e_ Emissions of Cancelled Tests, g
Urine MCS	2,549/2,904	52,382	35,483	209	
Fecal multiplex PCR	811/683	34,914	23,651	67.7	
Hepatitis C PCR	25/35	4,506	3,052	3.2	
EBV serology	106/187	3,037	2,057		17,711
CMV serology	93/186	2,697	1,827		
Hepatitis B PCR	18/25	2,738	1,855		
Total	3,602/4,020	100,274	67,925	279.9	
Labor of information technology staff to build and validate alerts				40 h	
Labor of medical microbiologists to determine resting time frames and alert comments				Part of routine duties	

Note. MCS, microscopy, culture, and sensitivity; PCR, polymerase chain reaction; EBT, Epstein-Barr virus; CMV, cytomegalovirus.

The 3,602 cancelled tests resulted in a financial cost saving of AU$100,274. The cost of waste saved by weight was calculated to be 280 kg during this 6-month period (209 kg for urine pots, 67.7kg for feces pots, and 3.2 kg for hepatitis C PCR/hepatitis B PCR/EBV serology and CMV serology tubes). The 199 cancelled EBV and CMV serology tests resulted in savings of 17,711 g of CO_2_e emissions for sample collection consumables, equivalent to driving 115 km in a standard car (Table 2).

The labor required for information technology staff to build and validate the alerts for the 6 tests studied was calculated to be 40 hours. The time required for the medical microbiologists to determine the retesting time frames and alert comments was not included as this work was considered as part of routine work and was partly performed for the test-rejection process that was in place prior to the duplicate alert implementation.

## Discussion

Customized alerts issued at the time of test ordering can have an enormous impact on reducing cost, waste, and inappropriate testing. Our customized alerts were only triggered if the test was ordered within a prespecified time frame determined according to the test type. Alerts also contained information about appropriate reordering frequency or indications for retesting based on test type. Our alerts also stated when the previous test was requested. Because our alerts are not merely a simple general alert but are tailored to the test type, they are also a form of decision support.

Concern has been raised that “alert fatigue” may decrease the effectiveness of these types of interventions over time. However, we examined a period of 16 months after alert implementation and found that the duplicate alerts remained very powerful because approximately half of duplicate requests were cancelled after the alert was triggered.

The cost of pathology testing includes the financial cost to the laboratory as well as the cost to the wider community when government funding is used to reimburse the healthcare institution. We used Australian Medicare reimbursements to measure costs that essentially represent cost to the public rather than laboratory costs to perform the test. A large proportion of healthcare costs in Australia are for pathology services. During the 2020–21 financial year, 18.4% of all Medicare spending (AU$5.36 billion or US$3.45 billion) was for pathology services.^23^ A 15-year meta-analysis concluded that 12%–44% of laboratory tests ordered globally are not clinically indicated.^24^ Thus, reducing unnecessary testing can yield significant savings.

The cost of pathology waste not only includes the financial cost to the healthcare institution of waste collection and disposal but also includes the cost to the greater community of the environmental impact of loss of land use, loss of biodiversity, and contamination of waterways. In a recent Australian study, the carbon footprint of common pathology tests was dominated by those of sample collection. Reducing unnecessary testing was thought to be the most effective approach to reducing the carbon footprint of pathology testing.^17^ We used calculations from this study to estimate the carbon footprint of sample collection consumables for EBV and CMV serology tests because sample collection consumables were determined to be the main sources of CO_2e_ emissions. However, the total carbon footprint savings of our alert implementation is likely to be much higher if other components of the test cycle (eg, tube and reagent manufacture, pneumatic tube system use, testing) are included and if the other tests are included particularly bearing in mind that the volume of the urine and feces pots is much greater than that of the blood containers.

Many laboratories without the facilities for electronic alerts at the point of test ordering have other processes in place to reduce duplicate testing. These processes are usually activated once a specimen has been received in the laboratory, which results in additional labor for the preanalytical steps of specimen collection, transport, access, and storage, as well as laboratory staff training regarding details of test rejection, including the time to inform clinicians of test rejection. Prior to implementation of duplicate order alerts, our laboratory had in place a process in which EBV and CMV serology testing was not performed for requests within 7 days of a previous test or for patients with IgG previously detected. Instead, a report was issued to alert the requester that the test was not performed. Because this process was manual, it was inconsistently applied, particularly due to the extra workload on staff to look up previous results, to issue the correct report, and to store the sample compared to the workload of simply doing the test. Additionally, the workload of receiving a call from the (often frustrated) requester and retrieving a stored specimen if repeat testing was clinically indicated was an extra barrier for adherence to this test nonperformance protocol. Significant further cost savings are likely when reduction of repeat testing is implemented prior to a sample being received in the laboratory. Due to their complexity, we did not measure costs savings associated with these preanalytical steps. Test cancellation by clinicians at the point of ordering is a more efficient and robust method of reducing duplicate testing and allows the valuable resource of laboratory staff to be used more wisely.

Calls to reduce inappropriate diagnostic testing have suggested that computerized ordering systems can improve ordering practice by providing real-time feedback and guidance on test appropriateness.^25^ In addition, when the decision to cancel a test is made by the clinical team rather than by laboratory staff, it is more likely to be accepted because the process is less authoritarian or confrontational for reducing waste. Behavioral approaches to guide decision making through the strategic placement of choice architecture while still maintaining prescriber autonomy are called “nudging strategies” and have been used successfully in altering the antibiotic selection of the prescriber.^26^ Information in the alert text is also an opportunity to educate requesting clinicians about appropriate reordering time frames and indications. This process is likely to promote a more collegial “team effort” approach to appropriate test ordering. Furthermore, because pathology test ordering has been shown to change over the course of a medical practitioner’s career and may increase significantly during the first 2 years of clinical practice,^27^ it may be particularly beneficial to utilize these educational activities to foster good ordering practices early in a clinician’s career during training in the hospital system.

Choosing Wisely Australia is part of a global healthcare initiative to promote a national dialogue on unnecessary tests, treatments, and procedures. Our pathology program at Monash Health was the champion organization of the inaugural Choosing Wisely Awards in 2022, and our EMR alerts were part of this testing optimization project.^28^

For our study, we only calculated cost savings of duplicate alert implementation for the 4 most ordered microbiology tests and the 2 most commonly ordered expensive tests. Because we did not calculate cost savings for the other 77 microbiology tests for which duplicate alerts were implemented, the total cost savings of alert implementation will likely be much greater. Currently in our institution, EMR is only implemented for inpatients. We expect that broader implementation of EMR with duplicate alerts to the outpatient setting will maximize the benefit. This added benefit may particularly affect the less frequently ordered but more expensive tests such as hepatitis B and hepatitis C PCR tests, which are ordered in greater number through the outpatient setting. The 2023 guidelines from the Society for Healthcare Epidemiology of America Diagnostic Stewardship Task Force mention restriction of repeat testing for *C. difficile* and pneumonia multiplex panel, but we have found that restriction of duplicate testing for many more tests can be a very powerful tool for diagnostic stewardship.^29^ Our study was a single-center study with 1 laboratory serving 3 teaching hospital campuses; further studies are needed in other laboratories to elucidate wider applicability in other situations.

We calculated the direct cost to the hospital of information technology staff labor to implement the duplicate alerts. This cost is only an initial financial outlay without any requirement for ongoing maintenance costs. To estimate the cost of waste by weight we estimated container volume to measure the waste disposal savings, but we acknowledge that this estimation may be affected by the fact that most containers are not completely filled on specimen submission and this is only an approximate estimate. However, this novel but important measurement has not been previously studied. We did not quantify indirect costs to the hospital such as potential harm of test nonperformance; for example, the yield of repeated *C. difficile* testing is low but not zero. However, our alerts are “soft stops” (ie, the requester still has the option to the request the test after reading the alert), so this risk is low.

Another limitation of our study was that cost for EBV and CMV serology calculations have assumed that only IgG and IgM are tested, which underestimates the true cost savings. For example, algorithms for testing in some laboratories states that if IgM is detected, CMV IgG avidity or EBV nuclear antigen IgG antibody are performed, with a recommendation for repeat serology. Furthermore, potential further cost savings of cascade testing, particularly in the case of false-positive IgM results, are not included here. Lastly, we did not measure the impact of alerts on clinician workload, nor did we assess the magnitude of the benefits of reducing repeat testing on patient health outcomes, comfort, and satisfaction. Although these aspects are more difficult to measure, they are nonetheless important to consider when implementing CCDS interventions.

In conclusion, we conclude that customized electronic duplicate order alerts issued at the time of request ordering can have an enormous impact on reducing the detrimental economic, health, and environmental effects of unnecessary testing. Further studies are required to ascertain the wider effects of such initiatives beyond financial gains. Institutional support for sophisticated digital solutions to optimize pathology testing can facilitate this endeavor.

## Supplementary material

For supplementary material accompanying this paper visit https://doi.org/10.1017/ice.2023.198. click here to view supplementary material
