# Supplementary material for: Impact of customized electronic duplicate order alerts on microbiology test ordering: Financial and environmental cost savings
Source: Infect Control Hosp Epidemiol. 2023 Oct 27;45(3):343–50. doi: 10.1017/ice.2023.198 (PMC10933501; doi:10.1017/ice.2023.198)
Supplement: Supplementary file 1 [file S0899823X23001988sup001.docx]

**Supplementary table 1. Weight of specimen collection containers**

|  | Weight Container 1 (grams) | Weight Container 2 (grams) | Weight Container 3 (grams) | Average weight (grams) |
| --- | --- | --- | --- | --- |
| Urine container (Sarstedt 75.9922.721) | 12.09 | 11.88 | 12.03 | 12.0 |
| Faeces container (Sarstedt 80.9924.027) | 13.47 | 13.51 | 13.38 | 13.45 |
| BD Vacutainer PPT (362791) | 8.07 | 7.97 | 8.05 | 8.03 |
| BD Vacutainer SST (367954) | 8.04 | 8.05 | 8.07 | 8.05 |
